# Investigation into Mass Loading Sensitivity of Sezawa Wave Mode-Based Surface Acoustic Wave Sensors

**DOI:** 10.3390/s130202164

**Published:** 2013-02-06

**Authors:** Ajay Achath Mohanan, Md Shabiul Islam, Sawal Hamid Md Ali, R. Parthiban, N. Ramakrishnan

**Affiliations:** 1 Electrical and Computer Systems Engineering, School of Engineering, Monash University Sunway Campus, Jalan Lagoon Selatan, Bandar Sunway 46150, Malaysia; E-Mails: ajay.mohanan@monash.edu (A.A.M.); rajendran.parthiban@monash.edu (R.P.); 2 Institute of Microengineering and Nanoelectronics (IMEN), Universiti Kebangsaan Malaysia (UKM), Bangi 43600, Malaysia; E-Mail: shabiul@ukm.my; 3 Department of Electrical Engineering, Electronics & Systems, Faculty of Engineering & Built Environment, Universiti Kebangsaan Malaysia (UKM), Bangi 43600, Malaysia; E-Mail: sawal@eng.ukm.my

**Keywords:** surface acoustic wave devices, Sezawa wave mode, mass loading effect, sensors, FEM simulation

## Abstract

In this work mass loading sensitivity of a Sezawa wave mode based surface acoustic wave (SAW) device is investigated through finite element method (FEM) simulation and the prospects of these devices to function as highly sensitive SAW sensors is reported. A ZnO/Si layered SAW resonator is considered for the simulation study. Initially the occurrence of Sezawa wave mode and displacement amplitude of the Rayleigh and Sezawa wave mode is studied for lower ZnO film thickness. Further, a thin film made of an arbitrary material is coated over the ZnO surface and the resonance frequency shift caused by mass loading of the film is estimated. It was observed that Sezawa wave mode shows significant sensitivity to change in mass loading and has higher sensitivity (eight times higher) than Rayleigh wave mode for the same device configuration. Further, the mass loading sensitivity was observed to be greater for a low ZnO film thickness to wavelength ratio. Accordingly, highly sensitive SAW sensors can be developed by coating a sensing medium over a layered SAW device and operating at Sezawa mode resonance frequency. The sensitivity can be increased by tuning the ZnO film thickness to wavelength ratio.

## 1. Introduction

Surface acoustic wave (SAW) devices employ mechanical waves such as Rayleigh waves, shear horizontal waves (SH), SH-acoustic plate mode and flexural plate mode for their device operation. SAW devices are widely used in telecommunications, sensors and actuator systems. These devices consist of metallic interdigital transducers (IDT) fabricated over piezoelectric substrates to excite and receive acoustic waves. SAW devices used in sensor applications usually involve a sensing medium made of a thin film coated over the acoustic path. Mass loading effect caused by the film is one of the principal sensing phenomenons in these types of sensors. In general mass loading of a thin film alters velocity of the acoustic wave as given by the relation shown below:

(1) $$\Delta v=(k_{1}+k_{2})\;\text{f v h}\;\rho -k_{2}\;\text{f v h}\;\left\{\frac{4\mu^{\prime }(\lambda^{\prime }+\mu^{\prime })}{v^{2}(\lambda^{\prime }+2\mu^{\prime })}\right\}$$

where *f* is the operating frequency without the film, *ρ* is the density of the film, *λ*′ and *µ* ′ are bulk and shear elastic moduli of the film, *v* is the velocity of SAW, and *h* is the film thickness. *k*_1_ and *k*_2_ are material constants of the substrate [1]. This change in velocity can be measured as a resonance frequency shift of the SAW device [2,3]. There has been great interest in improving the mass loading sensitivity in SAW devices by identifying suitable sensing films or device configurations and to thus develop highly sensitive SAW sensors. The following researchers have reported on improving or studying the mass loading sensitivity in SAW sensors and systems through experiments and computer aided simulations: Tsai *et al.* [4] proposed suitable methodology to design SAW sensors working on mass loading principle based on finite element method (FEM) simulation. This type of computer-aided design methodology helps in reducing the design and development costs and increases the robustness of the SAW sensor performance [4]. Kshetrimayum *et al.* [5] studied the influence of phase point operation of the SAW resonator sensor and reported that mass sensitivity can be enhanced if a SAW device has a non-linear dependence on the operating frequency. Fan *et al.* [6] reported an optimization technique for designing higher sensitivity layered and non-layered SAW sensors by identifying right operating frequencies, structural parameters and the properties of the multilayered materials used in these sensors. Bhasker *et al.* [7] studied the mass loading sensitivity of a ZnO/Si layered SAW device for different film stress, roughness, and crystalline size of the ZnO and concluded that sensor response can be tuned to obtain higher mass sensitivity. The study also suggest suitable processing conditions for the sensing layer on the SAW device substrate to obtain the desired sensing response of the SAW sensor [7]. More recently it is reported that the mass loading sensitivity in SAW sensors can be increased by employing high aspect ratio pillars as sensing medium in SAW sensors [8,9]. In this paper, we present higher mass loading sensitivity of a SAW sensor employing Sezawa wave mode for its device operation. Non-piezoelectric materials such as silicon, diamond are also used as substrates for SAW devices, where the substrate is coated with thin films made of piezoelectric materials such as zinc oxide and aluminium nitride. These devices fall in the category of layered SAW devices. Love waves are a type of acoustic waves propagating on layered SAW devices and several SAW sensors based on Love waves are reported [10,11]. Love waves do not have a vertical displacement component and are more sensitive to conductivity or permittivity changes making them well suited for liquid sensing applications. Sezawa waves are higher order SAW propagating on layered SAW devices where the acoustic velocity of the substrate is higher than that of the piezoelectric thin film deposited on it. Unlike Love waves, Sezawa waves have a vertical displacement component. The occurrence of Sezawa waves depends on the ratio of the piezoelectric thin film thickness to the wavelength of the SAW device [12]. Device applications of Sezawa waves have been explored by researchers in the past. The following are some examples of Sezawa wave mode-based SAW devices. Du *et al.* [12] reported a SAW microfluidic device and showed that Sezawa waves have better acoustic streaming than Rayleigh waves. Prechtel *et al.* [13] has developed a high frequency diamond-based SAW oscillator utilizing Sezawa wave mode. Weber *et al.* [14] observed and compared the propagation loss characteristics of Rayleigh and Sezawa wave modes travelling on the surface of a ZnO/SiO_2_/Si layered substrate. Talbi *et al.* [15] demonstrated a ZnO/Si layered SAW pressure sensor using Sezawa wave mode for device operation. FEM simulation of Sezawa waves in ZnO/Si SAW devices has also been reported in the past [16]. However, to the best of our knowledge, there is no study on the mass loading sensitivity comparison of these two modes in layered SAW devices. In this work we have performed FEM simulation of a ZnO/Si layered SAW device and investigated the mass loading sensitivity of Sezawa waves and verified their suitability to function as highly sensitive SAW sensors. In the initial stage of simulation, the Sezawa wave mode occurrence for two thicknesses of ZnO film (*h_ZnO_*) is investigated. Further, the resonance frequency shift caused by mass loading effect of a thin film on both Rayleigh and Sezawa waves are calculated from simulation results. Also the mass loading sensitivity of Sezawa waves is compared with a typical Love wave device with similar device geometry and configuration. The simulation methodology, results and discussion are presented in the following sections.

## 2. Simulation Methodology

**Figure 1.**
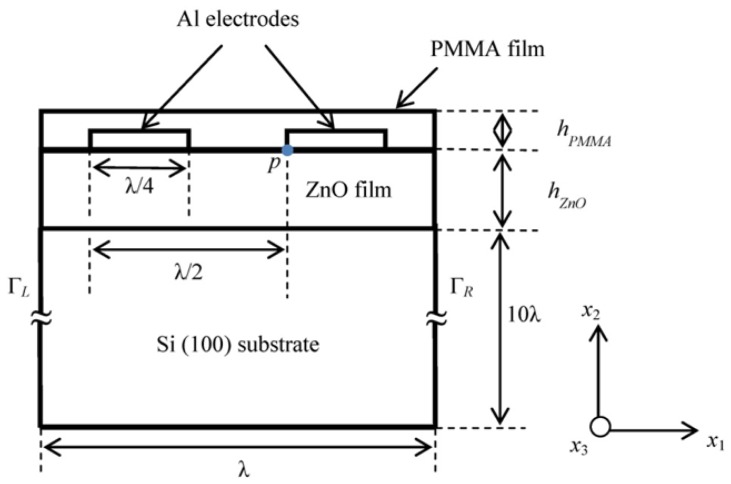
Geometry and boundaries of ZnO/Si layered one-port SAW resonator considered in the simulation.

FEM simulation of piezoelectric devices is well known and described elsewhere [17,18]. In this work we have performed FEM simulation of layered SAW device using the commercial COMSOL Multiphysics 4.2a, FEM software. A typical layered SAW resonator made of ZnO thin film coated over silicon substrate and an IDT with infinite number of finger pairs fabricated over ZnO film is investigated in the present study. Figure 1 shows the geometry of the layered SAW resonator considered in the simulation. It was decided to choose the dimensions of the device geometry such that the typical Rayleigh wave resonance frequency of the device in the range of 700 MHz to 1 GHz for the resonator. Accordingly a pitch length of 2 µm (λ/2) is chosen and ZnO film thickness is varied between ranges of 0.18 µm to 1 µm. Table 1 shows the dimensions of the IDT and substrate. As the structure and electric potential boundaries of the fingers in the IDT are periodic, a segment of 1λ width is considered as the SAW resonator geometry and suitable periodic boundary conditions are provided to the right and left boundaries of the layered SAW device (See Figure 1). As the displacement amplitude of Rayleigh and Sezawa wave modes are more concentrated at the surface, for simplicity a substrate of 10λ in length is chosen for the simulation. Since the displacement amplitude along *x*_3_-axis is negligible for the both modes [3], a 3D geometry with plain strain conditions is applied by choosing zero displacement constraint along *x*_3_-axis.

Table 2 shows the material constants used in the simulation. An extremely fine tetrahedral mesh was employed for all the FEM simulations conducted on the structure. Density, Young's modulus (E) and Poisson's ratio of Si (100); and elastic constants, piezoelectric stress constants, and dielectric constants of c-axis oriented ZnO are provided as material constants to the Si and ZnO substrates. Aluminium is chosen as the IDT electrode material. Appropriate elastic constants of ZnO thin film is provided in the simulation. It should be noted that ZnO thin film constants are appreciably different from the ZnO bulk constants as mentioned in [19,20].

Initially eigenfrequency analysis of the layered SAW resonator is performed for different heights of ZnO thin film (*h_ZnO_*) and resonance frequency of the SAW resonator in both Rayleigh mode (*f_R_*) and Sezawa mode (*f_S_*) is recorded. In this way the occurrence of Sezawa wave mode and its dominance over Rayleigh wave mode is studied. In the next stage, a thin film of poly-methyl methacrylate (PMMA) is coated over the surface of the ZnO film and the resonance frequency of both modes with different heights (*h_PMMA_*) of the PMMA film is recorded.

PMMA is chosen as an arbitrary material to introduce mass loading on the surface of the ZnO film. Moreover, PMMA is a well-known photoresist and precise thickness of PMMA thin film can be realized using simple spin coating techniques. Further, the resonance frequency shift of both the modes caused by the mass loading of the PMMA film is calculated. The resonance frequency shift is estimated by calculating the difference in resonance frequency (*f_R_* and *f_S_*) of the SAW device before and after PMMA loading on the layered substrate. Also mass loading sensitivity is analyzed for different thickness of ZnO film and resonance frequency shift caused by mass loading are presented as a function of *hk* (*h_ZnO_* × 2*π*/*λ*). An attempt is made to compare the mass loading sensitivity of Sezawa wave and Love waves. A ZnO/SiO_2_/Si layered Love wave mode based SAW device is modeled and the simulation setup is described under Section 3.

## 3. Results and Discussion

**Figure 2.**
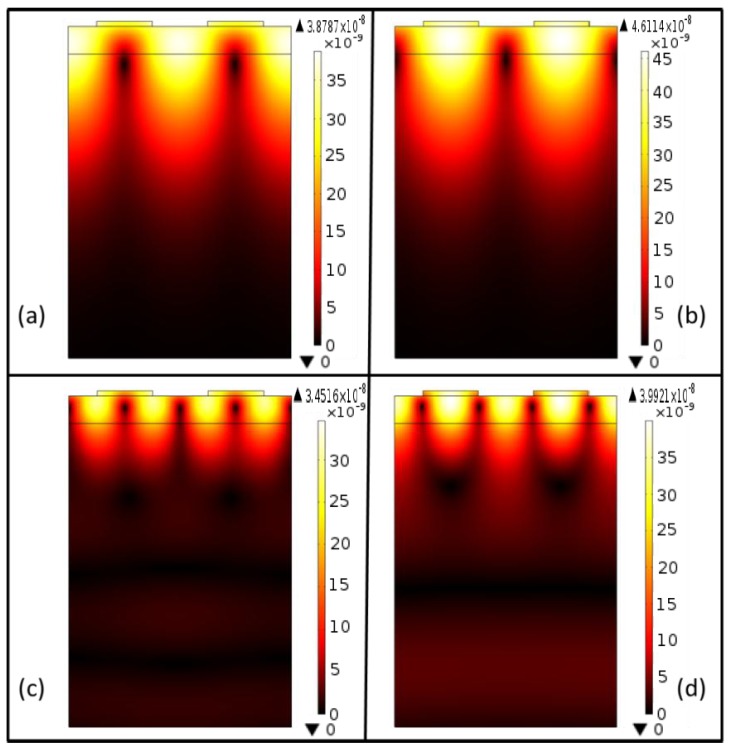
Total displacement profile showing acoustic wave mode shapes. (**a**) Rayleigh wave mode resonance (**b**) Rayleigh wave mode anti-resonance (**c**) Sezawa wave mode resonance (**d**) Sezawa wave mode anti-resonance.

Initially eigenfrequency analysis of the layered SAW resonator without mass loading is performed and total displacement (*t_d_*) profile is recorded for different heights of ZnO film (*h_ZnO_*). Total displacements of SAW is calculated by equation, $t_{d}=\sqrt{\left|u_{1}\right|{\;}^{2}+\left|u_{2}\right|{\;}^{2}+\left|u_{3}\right|{\;}^{2}}$ where *u*_1_, *u*_2_, and *u*_3_ are the particle displacement in *x*_1_, *x*_2_, and *x*_3_ directions (see Figure 1), respectively. The occurrence and resonance frequencies of Rayleigh and Sezawa mode SAW are identified from mode shapes of total displacement amplitude profile obtained for different eigenfrequencies. Figure 2 shows the *t_d_* profile of the SAW resonator structure at Rayleigh and Sezawa mode frequencies (resonance and anti-resonance) obtained for a typical ZnO film thickness of 0.5 µm. Figure 2(a,b) shows Rayleigh resonance and anti-resonance mode shapes observed at eigenfrequencies of 849.3 MHz and 858.5 MHz, respectively. Figure 2(c,d) shows typical Sezawa mode resonance and anti-resonance mode shapes observed at eigenfrequencies of 1.45 GHz, and 1.47 GHz, respectively. The mode shapes are similar to those observed in [16]. Further, frequency response of the SAW resonator is performed using the parametric solver of the COMSOL Multiphysics software to study the mode dominance at two lower ZnO film thickness, *h_ZnO_* = 0.1875 µm and 0.275 µm. An input voltage of 10 V is applied to the electrodes and *t_d_* at point *p* (see Figure 1) is recorded for a frequency sweep of 0 Hz to 1.8 GHz. It should be noted that *h_ZnO_* = 0.1875 µm (*hk* = 0.29, where *k* = 2π/*λ*) is the lowest ZnO thickness considered in the study, further lower thicknesses of ZnO film showed no acoustic wave modes due to low electromechanical coupling between the ZnO film and the Si substrate [12].

**Figure 3.**
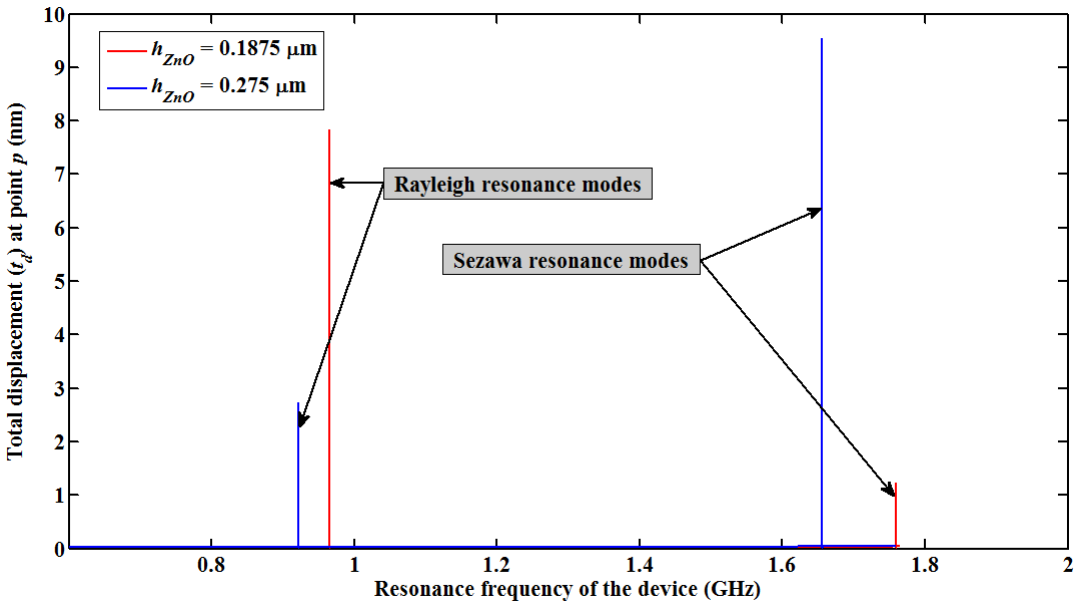
Plot of total displacement (*t_d_*) recorded at point *p* (see Figure 1) *versus* input frequency.

Figure 3 shows the plot of *t_d_versus* input frequency to the SAW resonator. The input frequencies for which the first and second substantial value of *t_d_* occurs are identified as *f_R_* and *f_S_* of the layered SAW resonator. It can be seen that for *h_ZnO_* = 0.1875 µm *f_R_* and *f_S_* are 966 MHz and 1.76 GHz, respectively; and for *h_ZnO_* = 0.275 µm *f_R_* and *f_S_* are 922 MHz and 1.66 GHz, respectively. It can be observed that for the lowest ZnO thickness of *h_ZnO_* = 0.1875 µm, total displacement observed for Rayleigh wave mode is six times higher than that of Sezawa wave mode, indicating the Rayleigh wave is dominant compared to Sezawa mode. However for the slightly higher thickness of *h_ZnO_* = 0.275 µm, Sezawa wave mode dominates over Rayleigh wave mode. Accordingly, it can also be seen that the resonance frequencies of both modes decrease as the thickness increases due to a reduction in the corresponding phase velocity. A lower thickness of ZnO film allows acoustic waves to penetrate deeper in to the Si substrate, thus more of the SAW energy is localized within the Si substrate and for higher thickness of the ZnO thin film SAW energy localizes within the ZnO layer thereby gradually reducing the corresponding phase velocity of the waves [12]. This part of the study also confirms the existence of Sezawa wave mode in a very thin film ZnO layer. It should be noted that the SAW velocity in Si is approximately 4,680 m·s^−1^ which is considerably higher than the ZnO velocity of 2,700 m·s^−1^[12].

**Figure 4.**
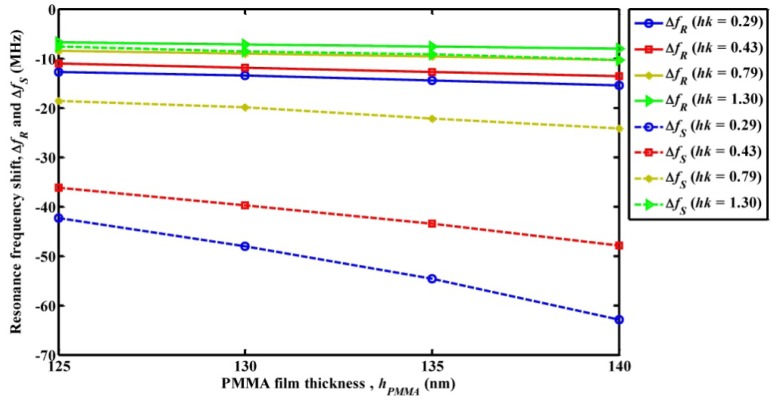
Plot of resonance frequency shift *versus* thickness of PMMA film (*h_PMMA_*).

To investigate the mass loading sensitivity of the Sezawa wave mode-based SAW device and compare the sensitivity with a Rayleigh wave mode SAW device, a thin film of polymethyl methacrylate (PMMA) is placed over the entire surface of the SAW resonator (see Figure 1). The height of the PMMA film (*h_PMMA_*) is varied from 125 nm to 140 nm in steps of 5 nm to simulate different mass loadings on the SAW resonator surface. Also to study the significance of *hk* on the mass loading sensitivity, four different thicknesses of ZnO film (*h_ZnO_*) are considered while maintaining a constant SAW wavelength. Accordingly, the sensitivity study is performed for *hk* values of 0.29, 0.43, 0.79 and 1.30, respectively. The electrode height of the SAW resonator is 100 nm, accordingly the lower limit of *h_PMMA_* is chosen as 125 nm. The upper limit *h_PMMA_* is chosen as 140 nm as further increase in *h_PMMA_* introduced attenuation to the acoustic waves for the case of lower *h_ZnO_*. Figure 4 shows the resonance frequency shift in Rayleigh wave mode resonance frequency (Δ*f_R_*) and Sezawa mode resonance frequency (Δ*f_S_*) *versus* different heights of PMMA film. From the figure it can be observed that for increase in *h_PMMA_* (increase in mass loading) the resonance frequency shift decreases for all cases of the study. It can be noted that Δ*f_s_* values are of higher magnitude compared to that of Δ*f_R_* for all four values of *hk* and *h_ZnO_* values examined. For instance, the Δ*f_R_* caused by mass loading of PMMA film of thickness *h_PMMA_* = 140 nm for *hk* = 0.29, 0.43, 0.79, and 1.3 is 15.44 MHz, 13.58 MHz, 10.35 MHz and 8.08 MHz respectively while the Δ*f_s_* observed for same case is 62.95 MHz, 47.87 MHz, 24.20 MHz and 10.29 MHz. It can also be seen that, as the *hk* value of the SAW device increases the difference between the resonance frequencies shift of both the modes decreases. Based on the observations from Figure 4, mass loading sensitivities for both modes for PMMA loading are summarized in Table 3. It can be observed from Table 3 that mass loading sensitivity increases for lower *hk* and the sensitivity of Sezawa wave mode is two to eight times greater than that of Rayleigh wave mode for all values of *hk* considered. A possible explanation for the higher mass loading sensitivity for Sezawa mode is as follows: the Sezawa waves are more localized in the Si substrate and hence the velocities of these waves are closer to the Si substrate velocity, while the Rayleigh wave mode velocity is comparatively lesser and closer to the ZnO substrate velocity [12]. In order to study the sensitivity of both wave modes to mass loading caused by thin film made of polymer material other than PMMA, the simulation is repeated for SU-8 film in place of PMMA in the simulation geometry (see Figure 1). It should be noted that SU-8 is well known for its rigidity and use in fabrication of high aspect ratio structures [8,21]. The material constants of SU-8 used in the simulation are shown in Table 2. The estimated mass loading sensitivity is tabulated in Table 4. It can be seen that the sensitivity results for case of SU-8 film are comparable to the results observed for case of a PMMA film as shown in Table 3.

**Figure 5.**
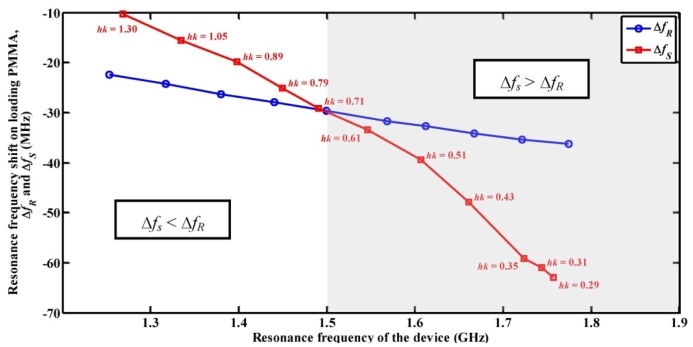
Plot of resonance frequency shift in Rayleigh and Sezawa wave mode (Δ*f_R_* , Δ*f_S_*) *versus* device operating frequency.

When the SAW device is operated at *f_s_*, an increase in mass loading decreases the higher Sezawa mode velocity and results in greater Δ*f_s_* compared to Δ*f_R_* observed when the device is operated at *f_R_*. It can also be noted that as the *hk* value is increased the resonance frequency shift for both modes decreases. Thus, a lower value of *hk* will provide higher mass loading sensitivity for both Rayleigh and Sezawa waves. The mass loading sensitivity of a Rayleigh wave SAW sensor depends on the operating frequency of the SAW device [22]. The present simulation study is extended to compare the sensitivity of the Sezawa wave based SAW device with Rayleigh wave based SAW device operating in a similar frequency range. Accordingly, FEM simulation of SAW resonator with Rayleigh wave resonance frequency greater than 1 GHz is performed and resonance frequency shifts obtained for both the cases are calculated. The pitch of the IDT is varied to obtain *f_R_* closer or equal to *f_s_* of SAW device with same *h_ZnO_*. Figure 5 shows Δ*f_s_* and Δ*f_R_* caused by an arbitrary mass of PMMA film *versus* the resonance frequency of the SAW resonator. The Δ*f_R_* trend is linear and has higher values than Δ*f_s_* for resonance frequencies below 1.5 GHz and *hk* = 0.71. It should be noted due to the difficulties in fabricating IDT patterns with sub-micro meter features (resolution less than 1 µm), realizing ultrahigh frequency Rayleigh wave devices on piezoelectric substrates is practically limited. It can also be seen from Figure 5 that Δ*f_s_* is higher than Δ*f_R_* for *hk* values less than 0.71 until 0.29. No wave modes could be detected for further decrease in *hk* value due to low electromechanical coupling between the ZnO film and the Si substrate [12]. Hence, Sezawa waves have higher mass loading sensitivity as compared to Rayleigh waves operating in the same frequency range for *hk* values less than 0.71.

**Figure 6.**
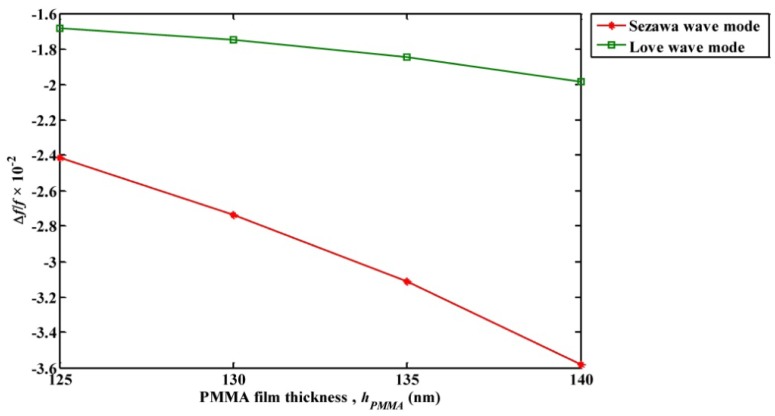
Plot of ratio of Δ *f*/*f versus* thickness of PMMA film (*h_PMMA_*). It should be noted that Δ*f*/*f* obtained for the wave modes are compared at their maximum sensitivity configuration. Accordingly the plots shown are obtained for Δ*f_S_*/*f_S_* at *hk* = 0.29 and Δ *f_L_*/*f_L_* at *h_ZnO_* = 0.21 µm.

Further the mass loading sensitivity of Sezawa wave compared with a typical layered ZnO/SiO_2_/Si Love wave mode SAW resonator. The geometry and device dimensions (pitch length of 2 µm) used for the simulation is same as shown in Figure 1, except that an additional SiO_2_ layer of 50 nm is introduced between the ZnO film and Si (100) Substrate. Initially, a PMMA thin film of thickness 140 nm is coated on the resonator surface and the resonance frequency shift caused by the mass loading of the PMMA film for different thickness of ZnO film (*h_ZnO_*) is recorded. It was observed that the maximum mass loading sensitivity occurred at *h_ZnO_* = 0.21 µm (0.0525λ) which is similar to the observations in [11]. Further the resonance frequency shift (Δ*f_L_*) caused by mass loading of different heights of PMMA film (*h_PMMA_*) at *h_ZnO_* = 0.21 µm is recorded and the ratio of Δ*f_L_* to the resonance frequency of the Love wave mode device, (Δ*f_L_*/*f_L_*) is calculated. The estimated Δ*f_L_*/*f_L_* is compared with Δ*f_S_*/*f_S_* obtained for Sezawa wave mode based SAW device at *hk* = 0.29. It should be noted from Figure 3 and Table 4 that the maximum mass loading sensitivity for Sezawa wave mode is obtained when *hk* = 0.29. Figure 6 shows a plot of the ratio of change in resonance frequency caused by PMMA film loading to the resonance frequency of the acoustic device (Δ*f*/*f*) observed for Sezawa wave mode and Love wave mode based SAW devices *versus* different thickness of PMMA film.

As an outline from Figure 6, it can be seen that Sezawa waves display higher sensitivity compared to Love waves. Also from Figure 6, it can be estimated that the change in Δ*f*/*f* per nanometer loading of PMMA film is 2.8 × 10^−4^ and 9.6 × 10^−4^ for Love wave mode and Sezawa wave mode respectively. Accordingly, Sezawa waves show approximately 3.4 times higher sensitivity than Love waves. Based on the investigations, it can be concluded that highly sensitive SAW sensors can be developed by depositing a sensing film over the layered ZnO/Si substrate of lower *hk* and operating the SAW device at Sezawa wave mode resonance frequency.

## 4. Conclusions

FEM simulation of a ZnO/Si layered SAW device is performed and mass loading sensitivity of Sezawa waves is investigated. Following are the derived conclusions from the study:

1. A SAW device employing Sezawa wave mode shows a decrease in resonance frequency for an increase in mass loading at the surface and exhibits significant mass loading sensitivity.
2. The mass loading sensitivity of Sezawa waves is two to eight times higher compared to Rayleigh waves propagating on the same SAW device.
3. Sezawa wave mode-based SAW devices with low values of *hk* show comparatively higher mass loading sensitivity.
4. In the simulation study, it is found that the Sezawa wave mode devices exhibit higher mass loading sensitivity as compared to a typical Love wave mode SAW device of similar configuration.

## Figures and Tables

**Table 1.** Dimensions of the IDT and substrate.

**Structure Property**	**Value**
Pitch of the electrodes (λ/2)	2 µm
Electrode width (λ/4)	1 µm
Electrode height	100 nm
Si substrate height (10λ)	40 µm

**Table 2.** Material constants used in the FEM simulation.

**Material**					**Elastic Constants**						**Piezoelectric Stress Constants**		

	Density kg/m3	E GPa	Poisson's Ratio	Relative Permittivity	*C*11	*C*12	*C*13	*C*33	*C*44	*C*66	*e*15	*e*31	*e*33

					GPa	GPa	GPa	GPa	GPa	GPa	C/m2	C/m2	C/m2
Si (100)	2,330	131	0.27	11.7	-	-	-	-	-	-	-	-	-
ZnO (c-axis oriented)	5,720	-	-	ε_11_ = 7.46	157	89	83	208	38	34	-0.45	-0.51	1.22
				ε_33_ = 8.59									
Aluminium	2,700	70	0.35	-	-	-	-	-	-	-	-	-	-
PMMA	1,190	3.0	0.40	3.0									
SU-8	1,190	4.02	0.22	3.0	-	-	-	-	-	-	-	-	-
SiO2	2,200	70	0.17	4.2	-	-	-	-	-	-	-	-	-

**Table 3.** Estimated mass loading sensitivities of Rayleigh and Sezawa waves for PMMA film loading.

**hk**	**Mass Loading Sensitivity of Rayleigh Waves**	**Mass Loading Sensitivity of Sezawa Waves**
**-**	**(MHz·nm^−1^)**	**(MHz·nm^−1^)**
0.29	0.19	1.66
0.43	0.17	0.86
0.79	0.14	0.40
1.30	0.09	0.22

**Table 4.** Estimated mass loading sensitivities of Rayleigh and Sezawa waves for SU-8 film loading.

***hk***	**Mass Loading Sensitivity of Rayleigh Waves**	**Mass Loading Sensitivity of Sezawa Waves**
**-**	**(MHz·nm^−1^)**	**(MHz·nm^−1^)**
0.29	0.21	1.03
0.43	0.18	0.68
0.79	0.14	0.43
1.30	0.09	0.24
